# Supplementary material for: The causal relationship between the human gut microbiota and pyogenic arthritis: a Mendelian randomization study
Source: Front Cell Infect Microbiol. 2024 Nov 26;14:1452480. doi: 10.3389/fcimb.2024.1452480 (PMC11629706; doi:10.3389/fcimb.2024.1452480)
Supplement: Supplementary file 1 [file DataSheet1.zip › mendelian test/ebi-a-GCST90027639.csv_leave_one_out.pdf]

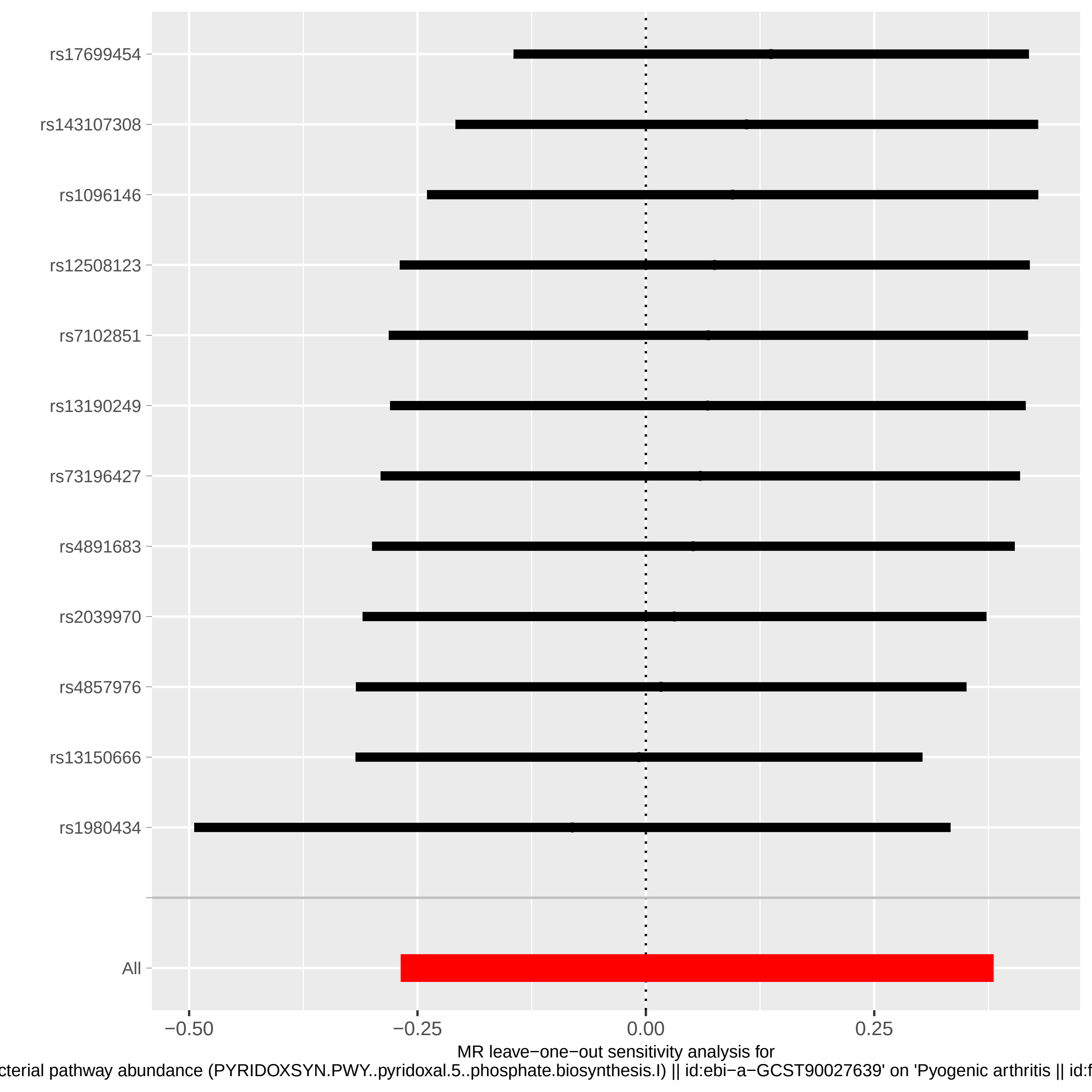
rs17699454
rs143107308
rs1096146
rs12508123
rs7102851
rs13190249
rs73196427
rs4891683
rs2039970
rs4857976
rs13150666
rs1980434
All
−0.50
−0.25
0.00
0.25
MR leave−one−out sensitivity analysis for
cterial pathway abundance (PYRIDOXSYN.PWY..pyridoxal.5..phosphate.biosynthesis.I) || id:ebi−a−GCST90027639' on 'Pyogenic arthritis || id:f
